# Associations Between 24-h Movement Behavior Composition and Muscle Health in Community-Dwelling Older Adults: The Kyotango Longevity Cohort Study

**DOI:** 10.3390/healthcare14142176

**Published:** 2026-07-19

**Authors:** Norikazu Hishikawa, Koshiro Sawada, Hironari Shinjo, Hiroki Kimura, Aya Yamanaka, Momoko Sakurai, Suzuyo Ohashi, Satoaki Matoba, Yasuo Mikami

**Affiliations:** 1 Department of Rehabilitation Medicine, Graduate School of Medical Science, Kyoto Prefectural University of Medicine, Kyoto 602-8566, Japan; hisikawa@koto.kpu-m.ac.jp (N.H.); hshinjo@koto.kpu-m.ac.jp (H.S.); h-k6850@koto.kpu-m.ac.jp (H.K.); ayahara@koto.kpu-m.ac.jp (A.Y.); momoima@koto.kpu-m.ac.jp (M.S.); s-ohashi@koto.kpu-m.ac.jp (S.O.); 2 Department of Rehabilitation, University Hospital, Kyoto Prefectural University of Medicine, Kyoto 602-8566, Japan; 3 Department of Longevity and Regional Epidemiology, Kyoto Prefectural University of Medicine, Kyoto 602-8566, Japan; matoba@koto.kpu-m.ac.jp; 4 Meiji University of Integrative Medicine, Kyoto 629-0392, Japan; y_mikami@meiji-u.ac.jp

**Keywords:** 24 h movement behaviors, compositional data analysis, accelerometry, muscle health, older adults

## Abstract

**Background/Objectives**: Daily movement behaviors are interdependent within a 24 h period. This study examined associations between 24 h movement behavior composition and muscle-related outcomes and estimated the effect of reallocating time to moderate-to-vigorous physical activity (MVPA) based on these outcomes. **Methods**: A total of 808 community-dwelling older adults from the Kyotango Longevity Cohort Study were included in this cross-sectional study. Waking behaviors (sedentary behavior, light-intensity physical activity, and MVPA) and sleep were assessed using accelerometers and sleep diaries. Daily time spent performing each behavior was averaged using the compositional mean to create a 24 h composition. Compositional data analysis and Firth’s penalized logistic regression were used to examine associations between movement behavior composition and muscle-related outcomes (low muscle strength, low muscle mass, and sarcopenia defined by the Asian Working Group for Sarcopenia 2025 criteria). Compositional isotemporal substitution analyses were performed to estimate time reallocations toward MVPA. **Results:** A higher relative contribution of MVPA was associated with lower odds of low muscle strength (odds ratio, 0.67; 95% confidence interval, 0.48–0.93; *p* = 0.016) and body mass index-adjusted low muscle mass (odds ratio, 0.81; 95% confidence interval, 0.66–0.99; *p* = 0.042). No associations were observed for height-adjusted low muscle mass and sarcopenia. Reallocating time from other waking activities toward MVPA was associated with lower odds of these outcomes, whereas no significant associations were observed for reallocations from sleep. **Conclusions:** A higher relative contribution of MVPA was associated with lower odds of low muscle strength and BMI-adjusted low muscle mass. Small reallocations of time from other waking activities toward MVPA may maintain muscle health in older adults.

## 1. Introduction

Daily time use is inherently constrained within a finite 24 h period; therefore, increases in one behavior necessarily displace time spent performing others [1,2]. Given this interdependence, the concept of 24 h movement behavior has received increasing attention [1,2]. This concept emphasizes that sleep, sedentary behavior (SB), light-intensity physical activity (LPA), and moderate-to-vigorous physical activity (MVPA) collectively influence a wide range of health outcomes, including frailty and mortality [1,3]. This perspective is particularly relevant in aging populations, where a substantial gap persists between lifespan and healthspan despite continued increases in life expectancy [4].

Sarcopenia, which is characterized by age-related declines in skeletal muscle mass and strength, represents a major public health challenge in aging societies [5]. It is strongly associated with functional decline, frailty, disability, and mortality [6] and is increasingly recognized as a key determinant of healthy aging [7]. Because declines in muscle mass and strength underlie the development of sarcopenia and its adverse consequences [8,9], preserving these components is essential for maintaining independence and healthy life expectancy in older adults. The updated Asian Working Group for Sarcopenia (AWGS) 2025 criteria retain the conventional height-adjusted muscle mass index and the newly introduced body mass index (BMI)-adjusted muscle mass index for assessing low muscle mass [5]. In addition, the AWGS 2025 consensus shifts its focus from sarcopenia alone to the broader concept of muscle health [5], highlighting the importance of investigating factors associated with muscle health.

Within the 24 h movement behaviors framework, MVPA is consistently associated with greater muscle strength and muscle mass [10]. However, two important limitations remain in the existing literature. First, large-scale epidemiological studies often rely on self-reported measures of physical activity [11], which are prone to recall and reporting bias [12]. Second, conventional analytical approaches generally treat movement behaviors as independent exposures and do not account for their interdependent and compositional nature within a fixed 24 h day.

Compositional data analysis (CoDA) addresses these limitations by modeling the relative distribution of time across movement behaviors while accounting for their inherent interdependence [13]. Moreover, compositional isotemporal substitution models enable an estimation of how reallocating time between behaviors is associated with health outcomes [14], providing a useful framework for interpreting movement behavior composition in observational studies. Previous isotemporal substitution studies have reported associations between physical activity and outcomes such as frailty and sarcopenia in older adults [15,16], primarily using non-compositional approaches. However, large-scale studies integrating objectively measured 24 h movement behaviors with CoDA remain limited, particularly for sarcopenia and broader muscle health outcomes. This gap limits our understanding of how the balance of daily movement behaviors relates to muscle health in older adults.

Japan, one of the most rapidly aging countries in the world [17], provides a unique context for investigating the determinants of healthy aging. Within Japan, the Kyotango area, a rural region in northern Kyoto Prefecture, has attracted attention as a potential model of successful aging, characterized by an advanced aging population (with approximately 38% of residents aged ≥65 years) and an exceptionally high proportion of centenarians (226 per 100,000 residents, which is approximately three times the national average) [18]. A previous study in this population showed that older adults engage in relatively high levels of MVPA [10], approximately twice the recommended levels [19]. These findings suggest that movement behavior patterns in this population may differ substantially from those observed in other settings. Therefore, the Kyotango population provides a unique opportunity to investigate how variations in movement behavior composition relate to muscle health within a highly active aging population.

The present study aimed to examine the associations between 24 h movement behavior and muscle health-related outcomes, including muscle strength, muscle mass, and sarcopenia, in community-dwelling older adults using objectively measured accelerometer data and CoDA. In addition, the potential effect of reallocating time from other movement behaviors to MVPA was evaluated using compositional isotemporal substitution models. This study extends previous research in this field [15,16] by applying the updated AWGS 2025 criteria [5], evaluating both height-adjusted and BMI-adjusted muscle mass definitions, and examining these associations in the distinctive Kyotango Longevity Cohort using objectively measured 24 h movement behavior data.

## 2. Materials and Methods

### 2.1. Study Design and Participants

This cross-sectional study used data from the Kyotango Longevity Cohort Study, an ongoing community-based prospective cohort study conducted by the Department of Longevity and Regional Epidemiology at Kyoto Prefectural University of Medicine (Kyoto, Japan). A total of 1258 community-dwelling adults aged ≥65 years who participated in health examinations between August 2017 and November 2025 were enrolled in the study. Participants were recruited through the municipal longevity health check-up program. Study information was disseminated on the official Kyotango City website, and community newsletters were distributed at Kyotango City Yasaka Hospital.

The study protocol was approved by the Ethics Review Board of Kyoto Prefectural University of Medicine (Approval No. ERB-C-885-9), and the study was conducted in accordance with the Declaration of Helsinki. Written informed consent was obtained from all participants. This study was reported in accordance with the Strengthening the Reporting of Observational Studies in Epidemiology guidelines [20].

### 2.2. Data Collection

Demographic information (age and sex) was obtained during health examinations. Anthropometric measurements (height and weight) were measured by trained staff using standardized protocols. BMI was calculated as weight in kilograms divided by the square of height in meters (kg/m^2^).

### 2.3. Assessment of 24 h Movement Behaviors

The 24 h day was partitioned into four movement behaviors: SB, LPA, MVPA, and sleep. Waking behaviors (SB, LPA, and MVPA) were assessed using a triaxial accelerometer (ActiGraph wGT3X-BT; ActiGraph LLC, Pensacola, FL, USA), which is widely used for physical activity assessment [21,22]. Participants were instructed to wear the device on the right hip for seven consecutive days and to remove it only during water-based activities. Acceleration was recorded at 30 Hz and processed using ActiLife software (version 6.13.4; ActiGraph LLC, Pensacola, FL, USA). Data were aggregated into 60 s epochs using the low-frequency extension filter to improve detection of low-intensity movements that are common among older adults [23]. Non-wear time was defined as ≥90 consecutive minutes of zero counts [24]. A valid day needed ≥10 h of accelerometer wear time during waking hours, and participants with ≥4 valid days [25], including at least one weekend day [26], were included in the analysis. Sleep periods were primarily determined using participant sleep diaries. A fixed sleep window (23:00–06:00) was applied only when diary data were unavailable [27]. Each 24 h period was divided into sleep and waking time. During waking time, activity intensity was classified as SB (<200 counts per minute [cpm]), LPA (200–2689 cpm), and MVPA (≥2690 cpm) based on established vector magnitude cut-points [28,29] corresponding to ≤1.5, 1.6–2.9, and ≥3.0 metabolic equivalents, respectively [30]. For each participant, mean daily durations of SB, LPA, MVPA, sleep, and non-wear time were calculated across valid days. Non-wear time was excluded from waking time and subsequently redistributed proportionally across the three waking behaviors while leaving sleep duration unchanged to generate a four-part 24 h (1440 min/day) movement behavior composition for compositional data analysis.

### 2.4. Muscle Outcomes

Muscle outcomes included muscle strength, muscle mass, and sarcopenia. Handgrip strength was measured using a hydraulic dynamometer (Jamar SH5001; SAKAI Medical Co., Ltd., Tokyo, Japan) with the arm hanging naturally at the side and the elbow flexed at 90°. The maximum value from two trials for each hand was used for analysis. Appendicular skeletal muscle mass (ASM) was assessed using bioelectrical impedance analysis (InBody 770; InBody Co., Ltd., Seoul, Republic of Korea). Height-adjusted ASM (kg/m^2^) and BMI-adjusted ASM were calculated by dividing ASM (kg) by height squared (m^2^) and BMI, respectively. Both indices are included in the updated AWGS 2025 criteria for assessing low muscle mass [5]. Because height-adjusted and BMI-adjusted ASM are normalized to height and BMI, respectively, the two indices may reflect different aspects of muscle mass. Low muscle strength, low muscle mass, and sarcopenia were defined according to the AWGS 2025 criteria cut-off values [5]. Low muscle strength was defined as <28 kg for men and <18 kg for women. Low muscle mass was defined as a height-adjusted ASM < 7.0 kg/m^2^ for men and <5.7 kg/m^2^ for women, and a BMI-adjusted ASM < 0.83 for men and <0.57 for women. Sarcopenia was defined as the coexistence of low muscle strength and low muscle mass.

### 2.5. Statistical Analyses

Participant characteristics are presented as the mean (standard deviation) for continuous variables and numbers (percentages) for categorical variables. Differences between sexes were assessed using the Mann–Whitney U test for continuous variables and Fisher’s exact test for categorical variables. Age was categorized as 65–74, 75–84, and ≥85 years. The prevalence of low muscle strength, low muscle mass, and sarcopenia was calculated by age group and sex. Trends across age groups were assessed using the Cochran–Armitage trend test.

Time-use data were expressed as proportions of the 24 h day and analyzed using CoDA. The composition was transformed into isometric log-ratio (*ilr*) coordinates to account for the interdependence of movement behaviors [31]. To examine the relative contribution of MVPA, *ilr* coordinates were constructed based on a predefined sequential binary partition. MVPA was placed first to interpret its relative contribution to the overall composition [2]. Given the four-part composition of movement behaviors, three *ilr* coordinates were derived: the first (*ilr*_1_) represents MVPA in relation to the remaining behaviors, the second (*ilr*_2_) represents LPA in relation to SB and sleep, and the third (*ilr*_3_) represents SB in relation to sleep. The *ilr* coordinates are defined as follows:

$$ilr_{1}=\sqrt{\frac{3}{4}}\ln\left(\frac{MVPA}{\sqrt[{3}]{SB\times LPA\times sleep}}\right)$$

$$ilr_{2}=\sqrt{\frac{2}{3}}\ln\left(\frac{LPA}{\sqrt{SB\times sleep}}\right)$$

$$ilr_{3}=\sqrt{\frac{1}{2}}\ln\left(\frac{SB}{sleep}\right)$$

Higher values of an *ilr* coordinate indicate that relatively more time is spent on the movement behaviors represented by that coordinate than on other behaviors. For example, an odds ratio < 1 for *ilr*_1_ indicates that spending more time on MVPA compared with other movement behaviors is associated with lower odds of adverse muscle-related outcomes.

Multivariable logistic regression models were used to examine associations between movement behavior composition and muscle-related outcomes. Given the relatively low number of outcome events for certain conditions, particularly sarcopenia (36 and 39 events for height- and BMI-adjusted definitions, respectively), Firth’s penalized logistic regression was used to reduce small-sample bias and improve estimation stability. Models were adjusted for age and sex. Other potential confounders were intentionally excluded from the primary models to avoid over-adjustment and preserve model stability, particularly given the relatively small number of outcome events for some outcomes. Anthropometric variables (height, weight, and BMI) were not included because muscle mass outcomes were calculated using these variables. Adjusting for them could introduce mathematical coupling and lead to over-adjustment. In addition, compositional isotemporal substitution analyses were conducted to estimate the effect of reallocating time to MVPA from SB, LPA, and sleep in 5 min increments (−30 to +60 min). Predicted odds ratios for each outcome were derived from regression models, and 95% confidence intervals (CIs) were estimated using bootstrap resampling (10,000 iterations).

All analyses were performed using R version 4.5.2 (R Foundation for Statistical Computing, Vienna, Austria). CoDA was conducted with the compositions package (version 2.0.9), penalized logistic regression with the logistf package (version 1.26.1), and bootstrap analyses with the boot package (version 1.3.32). A two-sided *p*-value < 0.05 was considered statistically significant.

## 3. Results

The participant selection process is illustrated in Supplementary Figure S1. A total of 1258 community-dwelling older adults participated in the Kyotango Longevity Cohort Study. Of these, 420 participants did not complete the 24 h movement behavior assessment. In total, 15 participants with insufficient valid accelerometer data (13 with fewer than four valid days and 2 without a weekend day), 7 participants with missing body composition data, and 8 participants with lower-limb dysfunction were excluded. Participants with lower-limb dysfunction were excluded because such conditions could affect physical activity measurements and muscle-related assessments. Consequently, 808 participants were included in the final analysis. Sleep diary data were available for 747 participants (92.5%), whereas the remaining 61 participants (7.5%) without sleep diary data were retained in the analysis. Table 1 presents the demographic and clinical characteristics of the study participants. The mean age was 74.0 years, and 486 participants (60.1%) were women. Men were older and had greater height, weight, and BMI than women (all *p* < 0.001). Handgrip strength and ASM, including both height-adjusted and BMI-adjusted indices, were also higher in men than in women (all *p* < 0.001).

Table 2 shows the overall and sex-specific prevalences of low muscle strength, low muscle mass, and sarcopenia. The prevalence of low muscle strength was 7.1%, the prevalence of low muscle mass was 36.1% according to height-adjusted ASM and 32.3% according to BMI-adjusted ASM, and the prevalence of sarcopenia was 4.5% according to height-adjusted ASM and 4.8% according to BMI-adjusted ASM. Sex differences were observed for selected outcomes, particularly height-adjusted low muscle mass.

Age-specific prevalences are presented in Table 3, demonstrating significant increases in the prevalence of low muscle strength, low muscle mass, and sarcopenia with advancing age (all *p* for trend <0.001).

The compositional mean of the 24 h movement behaviors was 473.2 min/day for SB (511.1 for men and 447.9 for women), 467.9 min/day for LPA (415.2 and 504.5), 33.5 min/day for MVPA (34.9 and 32.5), and 465.4 min/day for sleep (478.8 and 455.0). Table 4 shows the associations between the *ilr* coordinates representing the 24 h movement behavior composition and muscle-related outcomes. A higher relative contribution of MVPA (*ilr*_1_) was significantly associated with lower odds of low muscle strength (odds ratio, 0.67; 95% CI, 0.48–0.93; *p* = 0.016) and BMI-adjusted low muscle mass (odds ratio, 0.81; 95% CI, 0.66–0.99; *p* = 0.042). However, no significant associations were observed for height-adjusted low muscle mass or sarcopenia (height- or BMI-adjusted). In addition, neither *ilr*_2_ nor *ilr*_3_ was significantly associated with any muscle-related outcome.

**Figure 1:**
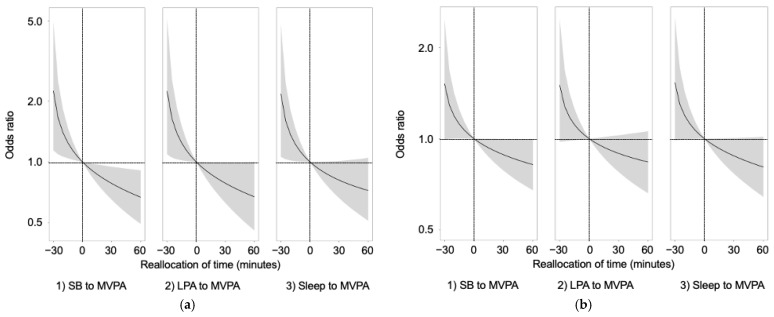
Compositional isotemporal substitution analyses of the associations between reallocating time to MVPA and muscle-related outcomes. (**a**) Low muscle strength. (**b**) BMI-adjusted low muscle mass. BMI, body mass index; OR, odds ratio; SB, sedentary behavior; LPA, light-intensity physical activity; MVPA, moderate-to-vigorous physical activity. The plots show the model-estimated ORs for low muscle strength associated with reallocating time from SB, LPA, or sleep to MVPA. The x-axis represents the amount of time (minutes) reallocated to MVPA, with 0 min indicating no reallocation from the reference composition. The y-axis represents the ORs relative to the reference composition; the reference composition corresponds to the mean 24 h movement behavior composition of the study population. The solid lines represent the estimated ORs, and the shaded areas indicate 95% confidence intervals. The horizontal dashed line indicates an OR of 1.0 (indicating no difference from the reference composition), and the vertical dashed line represents the reference composition (0 min reallocated). Models were adjusted for age and sex. These curves represent model-estimated associations between different theoretical time allocations and the outcome for the constraint of a fixed 24 h day. They should not be interpreted as estimates of intervention effects.

The results of the compositional isotemporal substitution analyses are shown in Figure 1. Reallocating time from SB or LPA to MVPA was associated with lower odds of low muscle strength. For BMI-adjusted low muscle mass, only reallocations from SB to MVPA were associated with lower odds. As illustrated by the continuous non-linear curves, greater reallocations of time to MVPA were generally associated with progressively lower odds where statistically significant associations were observed. No significant associations were observed for reallocations from sleep to MVPA.

## 4. Discussion

This study provides evidence, based on objectively measured 24 h movement behaviors and CoDA, that a greater relative contribution of MVPA to daily activity is associated with lower odds of adverse muscle-related outcomes in community-dwelling older adults. Specifically, a higher relative proportion of MVPA was associated with lower odds of low muscle strength and BMI-adjusted low muscle mass. In addition, compositional isotemporal substitution analyses suggested that reallocating time from SB or LPA to MVPA was associated with reduced odds of unfavorable outcomes.

The prevalence of sarcopenia observed in this study (approximately 5%) was lower than that reported in previous studies of community-dwelling older adults, where estimates typically range from approximately 10% to 40% [32]. Studies conducted in Japanese populations have also reported higher prevalence estimates of approximately 7% to 10% [33], suggesting that the pervasiveness of sarcopenia in the present study is relatively low even within the Japanese context. This may reflect, at least in part, the distinctive characteristics of the Kyotango population, which include relatively high levels of physical activity [10] despite their older age profile. Nevertheless, the prevalence of low muscle strength, low muscle mass, and sarcopenia increased significantly with age, consistent with previous literature [34,35]. This pattern underscores the progressive nature of age-related declines in muscle health.

A notable finding of this study is that the observed associations were specific to certain muscle-related outcomes, particularly muscle strength and BMI-adjusted muscle mass. By contrast, no significant associations were observed for height-adjusted muscle mass or sarcopenia. Several explanations may account for this pattern. First, BMI-adjusted muscle mass incorporates body mass and performs comparably to or better than height-adjusted indices in identifying individuals with low muscle strength, functional impairment, and adverse outcomes [36,37,38,39]. By contrast, height-adjusted indices primarily reflect muscle mass relative to linear body size (e.g., height) rather than overall body composition. This distinction may be particularly important in Asian populations, who tend to have a higher body fat percentage and greater ectopic fat accumulation at a given BMI than Western populations [40]. Height-adjusted muscle mass may therefore not fully reflect muscle health in this population. Second, sarcopenia is a composite and binary outcome that may be less sensitive to differences in movement behavior than continuous or intermediate phenotypes. Third, the relatively low number of sarcopenia cases in this population may have limited statistical power to detect associations, despite the use of penalized regression methods. Therefore, the absence of a statistically significant association with sarcopenia should be interpreted with caution. Taken together, these findings suggest that while the strength of the observed associations differed for muscle mass definition, the BMI-adjusted index may be particularly informative for evaluating associations between movement behavior composition and muscle mass in older Asian populations.

Another finding was that significant associations were observed for the relative contribution of MVPA, whereas no significant associations were observed for the relative distribution among the other movement behaviors. This may suggest that the relative amount of time spent in MVPA, rather than how the remaining time is distributed among SB, LPA, and sleep, is more relevant to muscle-related outcomes, possibly because maintaining muscle strength and mass may require sufficient mechanical loading and neuromuscular stimulus. Furthermore, the compositional isotemporal substitution analyses provided complementary insights into model-based reallocations of time toward MVPA within the constraints of a 24 h day. Reallocating time from SB or LPA to MVPA was associated with lower odds of low muscle strength, whereas only reallocations from SB to MVPA were associated with lower odds of BMI-adjusted low muscle mass. This difference may reflect the smaller contrast in activity intensity between LPA and MVPA than between SB and MVPA. Taken together, the CoDA and isotemporal substitution analyses suggest that MVPA may play a particularly important role in muscle-related outcomes within the overall 24 h movement behavior composition. Although the optimal reallocation patterns differed by outcome, modest reallocations toward MVPA were associated with favorable findings for low muscle strength and BMI-adjusted low muscle mass. This is particularly relevant for older adults, who often face barriers to engaging in higher volumes of MVPA [41].

From a practical and public health perspective, these findings suggest that the relative balance of daily movement behaviors, not only the total volume of physical activity, may be relevant to muscle health in older adults [15]. Even modest differences in the relative contribution of MVPA within the 24 h day were associated with selected muscle-related outcomes. This perspective may be particularly pertinent for older adults, for whom adherence to recommended activity levels is low, given the high global prevalence of physical inactivity [42]. However, these associations should not be interpreted as evidence that altering movement behavior composition improves muscle health. Confirmation in longitudinal and intervention studies is needed before public health recommendations can be drawn.

Although this study was conducted in a specific regional population, these findings may have broader relevance for aging populations worldwide. The Kyotango population is characterized by relatively high levels of physical activity and preserved functional capacity despite advanced age. However, the relatively active characteristics of this population may have attenuated the observed associations and should be considered when interpreting the findings. The Kyotango population may nevertheless represent a population-based example of successful aging. Taken together, the present results suggest that the balance of daily movement behaviors, particularly the relative contribution of MVPA, may be relevant to maintaining muscle health in older adults. In addition, by applying the updated AWGS 2025 criteria [5], which emphasize muscle health as a multidimensional construct beyond skeletal muscle quantity, the present study provides contemporary and clinically relevant evidence on the associations between daily movement behavior composition and muscle health in older adults.

The present study has several limitations that should be acknowledged. First, the cross-sectional design precludes causal inference, and reverse causation cannot be ruled out; for example, low muscle strength or muscle mass may cause individuals to be less physically active, rather than the reverse. Accordingly, the observed associations should be interpreted as relationships rather than evidence of causal effects. Longitudinal studies are needed to determine whether changes in movement behavior composition precede changes in muscle health. Second, although hip-worn accelerometers provide objective measurements, they may not fully capture certain types of activity, such as cycling, water-based activities, and upper-body movements. Third, sleep periods were primarily determined using participant sleep diaries, and a fixed time window was applied only when diary data were unavailable. Although sensitivity analyses restricted to participants with sleep diary data yielded generally similar estimates (Supplementary Table S1), this approach may have introduced some measurement error. Fourth, the number of events for certain outcomes, particularly sarcopenia, was relatively small, which may have limited the statistical power despite our use of Firth’s penalized logistic regression. In addition, because multiple outcomes and compositional analyses were performed, the possibility of a type I error due to multiple comparisons cannot be excluded. Fifth, the primary models were intentionally adjusted only for age and sex to avoid over-adjustment and preserve model stability, particularly given the relatively small number of outcome events for some outcomes. Although sensitivity analyses with additional adjustment for available covariates, including smoking status, drinking status, educational attainment, marital status, and treatment for hypertension, diabetes, and dyslipidemia, yielded materially similar results (Supplementary Table S2), residual confounding from unmeasured factors cannot be excluded. For example, dietary intake, nutritional status, and medication use may affect muscle health [43,44], while other chronic health conditions may also have influenced the observed associations. Finally, the study population was drawn from a single rural region in Japan and consisted of relatively healthy and active older adults who participated in municipal health examinations. This may have introduced selection bias and limited the generalizability of our findings to frailer or more sedentary older adults and to populations living in urban areas, other countries, or different geographic, cultural, and healthcare settings.

## 5. Conclusions

A greater relative contribution of MVPA to the 24 h movement behavior composition was associated with lower odds of adverse muscle-related outcomes in community-dwelling older adults. Model-based reallocations of time from SB or LPA to MVPA were also associated with favorable muscle-related outcomes. However, because of the cross-sectional design, these findings represent associations and do not demonstrate that reallocating time to MVPA improves or maintains muscle health. Future longitudinal and intervention studies are needed to determine whether changes in movement behavior composition can improve muscle health and promote healthy aging.

## Figures and Tables

**Table 1:** Demographic characteristics of study participants.

	Total (*n* = 808)	Men (*n* = 322)	Women (*n* = 486)	*p*-Value
Age, years	74.0 (5.5)	75.0 (5.9)	73.3 (5.2)	<0.001
Age group, *n*				
65–74	490 [60.6]	175 [54.3]	315 [64.8]	0.003
75–84	289 [35.8]	129 [40.1]	160 [32.9]	0.038
≥85	29 [3.6]	18 [5.6]	11 [2.3]	0.013
Height, cm	157.4 (8.4)	164.7 (6.5)	152.5 (5.6)	<0.001
Weight, kg	56.5 (10.0)	63.5 (9.2)	51.8 (7.4)	<0.001
BMI, kg/m^2^	22.7 (3.0)	23.4 (2.9)	22.3 (3.0)	<0.001
Muscle strength				
Handgrip, kg	29.3 (8.4)	37.1 (7.0)	24.1 (4.2)	<0.001
Muscle mass				
ASM, kg	16.2 (3.9)	20.1 (3.0)	13.7 (1.9)	<0.001
Height-adjusted ASM, kg/m^2^	6.5 (1.0)	7.4 (0.7)	5.9 (0.6)	<0.001
BMI-adjusted ASM	0.72 (0.16)	0.86 (0.12)	0.62 (0.09)	<0.001
Movement behaviors				
SB, minutes/day	471.9 (112.1)	508.2 (119.6)	447.9 (100.0)	<0.001
LPA, minutes/day	466.7 (108.2)	414.7 (105.0)	501.2 (95.9)	<0.001
MVPA, minutes/day	46.0 (35.2)	50.1 (40.9)	43.3 (30.7)	0.136
Sleep, minutes/day	455.3 (64.7)	467.0 (68.2)	447.5 (61.0)	<0.001

BMI, body mass index; ASM, appendicular skeletal muscle mass; SB, sedentary behavior; LPA, light-intensity physical activity; MVPA, moderate-to-vigorous physical activity. Data are presented as means (standard deviation) or numbers [percentages]. Movement behaviors are presented as arithmetic means. *p*-values for sex comparisons were calculated using the Mann–Whitney U test for continuous variables and Fisher’s exact test for categorical variables.

**Table 2:** Overall and sex-specific prevalence of low muscle strength, low muscle mass, and sarcopenia.

	Total (*n* = 808)	Men (*n* = 322)	Women (*n* = 486)	*p*-Value
Low muscle strength	57 [7.1]	24 [7.5]	33 [6.8]	0.826
Low muscle mass				
Height-adjusted	292 [36.1]	95 [29.5]	197 [40.5]	0.002
BMI-adjusted	261 [32.3]	123 [38.2]	138 [28.4]	0.005
Sarcopenia				
Height-adjusted	36 [4.5]	16 [5.0]	20 [4.1]	0.688
BMI-adjusted	39 [4.8]	19 [5.9]	20 [4.1]	0.321

BMI, body mass index; ASM, appendicular skeletal muscle mass. Height-adjusted and BMI-adjusted indicate that low muscle mass and sarcopenia were defined using height-adjusted and BMI-adjusted ASM, respectively. Data is presented as the number [percentage]. *p*-values were calculated using Fisher’s exact test for sex comparisons.

**Table 3:** Age-specific prevalence of low muscle strength, low muscle mass, and sarcopenia.

	65–74 (*n* = 490)	75–84 (*n* = 289)	≥85 (*n* = 29)	*p* for Trend
Low muscle strength	18 [3.7]	31 [10.7]	8 [27.6]	<0.001
Low muscle mass				
Height-adjusted	171 [34.9]	102 [35.3]	19 [65.5]	<0.001
BMI-adjusted	112 [22.9]	128 [44.3]	21 [72.4]	<0.001
Sarcopenia				
Height-adjusted	10 [2.0]	18 [6.2]	8 [27.6]	<0.001
BMI-adjusted	7 [1.4]	26 [9.0]	6 [20.7]	<0.001

BMI, body mass index; ASM, appendicular skeletal muscle mass. Height-adjusted and BMI-adjusted indicate that low muscle mass and sarcopenia were defined using height-adjusted and BMI-adjusted ASM, respectively. Data are presented as the number [percentage]. *p* for trend values were calculated using the Cochran–Armitage trend test for age groups.

**Table 4:** Associations between 24 h movement behavior composition and muscle outcomes.

	Low Muscle Strength		Low Muscle Mass (Height-Adjusted)		Low Muscle Mass (BMI-Adjusted)		Sarcopenia (Height-Adjusted)		Sarcopenia (BMI-Adjusted)	
	OR (95% CI)	*p*-Value	OR (95% CI)	*p*-Value	OR (95% CI)	*p*-Value	OR (95% CI)	*p*-Value	OR (95% CI)	*p*-Value
*ilr*_1_	0.67 (0.48–0.93)	0.016	0.86 (0.71–1.05)	0.137	0.81 (0.66–0.99)	0.042	0.77 (0.52–1.15)	0.196	0.80 (0.54–1.20)	0.276
*ilr*_2_	1.22 (0.40–3.80)	0.729	0.74 (0.40–1.37)	0.333	0.84 (0.43–1.62)	0.604	0.59 (0.15–2.30)	0.443	1.50 (0.40–5.71)	0.552
*ilr*_3_	1.49 (0.35–6.30)	0.593	0.46 (0.21–1.01)	0.053	0.91 (0.39–2.09)	0.828	1.04 (0.17–6.31)	0.967	1.40 (0.25–7.70)	0.699

BMI, body mass index; ASM, appendicular skeletal muscle mass; *ilr*, isometric log-ratio; OR, odds ratio; CI, confidence interval. Height-adjusted and BMI-adjusted indicate that low muscle mass and sarcopenia were defined using height-adjusted and BMI-adjusted ASM, respectively. Models were adjusted for age and sex. *p*-values were obtained using Firth’s penalized logistic regression. *ilr* coordinates are isometric log-ratio transformations of the 24 h movement behaviors. *ilr*_1_ represents moderate-to-vigorous physical activity relative to the remaining behaviors; *ilr*_2_ represents light-intensity physical activity relative to sedentary behavior and sleep; and *ilr*_3_ represents sedentary behavior relative to sleep.

## Data Availability

The data that support the findings of this study are available from the corresponding author upon reasonable request.

## Supplementary Materials

The following supporting information can be downloaded at https://www.mdpi.com/article/10.3390/healthcare14142176/s1, Figure S1: Participant selection flowchart; Table S1: Sleep diary-restricted sensitivity analysis; Table S2: Sensitivity analysis with additional covariate adjustment.

## Abbreviations

ASM	Appendicular skeletal muscle mass
AWGS	Asian Working Group for Sarcopenia
BMI	Body mass index
CI	Confidence interval
CoDA	Compositional data analysis
ilr	Isometric log-ratio
LPA	Light-intensity physical activity
MVPA	Moderate-to-vigorous physical activity
OR	Odds ratio
SB	Sedentary behavior
