# Supplementary material for: Associations Between 24-h Movement Behavior Composition and Muscle Health in Community-Dwelling Older Adults: The Kyotango Longevity Cohort Study
Source: Healthcare (Basel). 2026 Jul 19;14(14):2176. doi: 10.3390/healthcare14142176 (PMC13409974; doi:10.3390/healthcare14142176)
Supplement: Supplementary file 1 [file healthcare-14-02176-s001.zip › healthcare-4427371-supplementary.pdf]

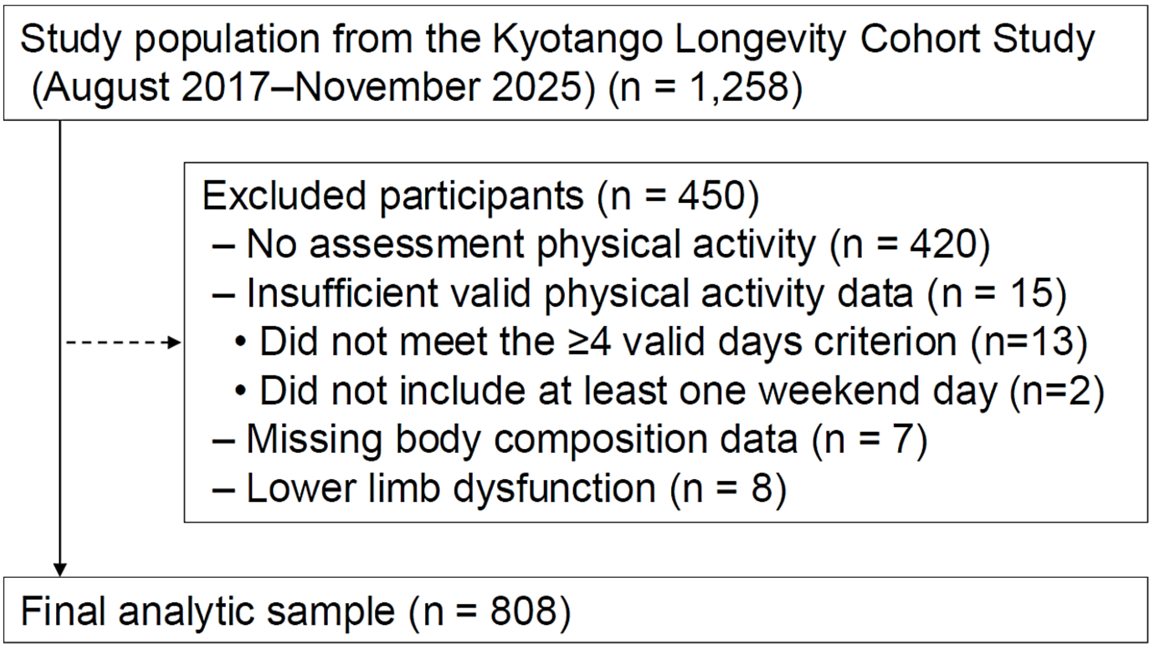


**Figure S1**. Participant selection flowchart.

**Table S1**. Comparison of the primary analysis and the sleep diary-restricted sensitivity analysis of the associations between the relative contribution of MVPA (*ilr₁*) and muscle-related outcomes.

| | Primary analysis (*n* = 808) | | Sensitivity analysis (*n* = 747) | |
|---|---|---|---|---|
| | **OR (95% Cl)** | ***P*-value** | **OR (95% Cl)** | ***P*-value** |
| Low muscle strength | 0.67 (0.48–0.93) | 0.016 | 0.70 (0.50–0.98) | 0.040 |
| Low muscle mass<br>Height-adjusted | 0.86 (0.71–1.05) | 0.137 | 0.85 (0.69–1.04) | 0.110 |
| BMI-adjusted | 0.81 (0.66–0.99) | 0.042 | 0.82 (0.66–1.01) | 0.062 |
| Sarcopenia<br>Height-adjusted | 0.77 (0.52–1.15) | 0.196 | 0.82 (0.55–1.26) | 0.353 |
| BMI-adjusted | 0.80 (0.54–1.20) | 0.276 | 0.81 (0.54–1.23) | 0.303 |

BMI, body mass index; ASM, appendicular skeletal muscle mass; *ilr*, isometric log-ratio; OR, odds ratio; CI, confidence interval. Height-adjusted and BMI-adjusted indicate that low muscle mass and sarcopenia were defined using height-adjusted and BMI-adjusted ASM, respectively. All models were fitted using Firth's penalized logistic regression and adjusted for age and sex. Sensitivity analyses were restricted to participants with sleep diary data.

**Table S2**. Comparison of the primary and sensitivity analyses with additional covariate adjustment of the associations between the relative contribution of MVPA (*ilr₁*) and muscle-related outcomes.

| | Primary analysis (*n* = 777) | | Sensitivity analysis (*n* = 777) | |
|---|---|---|---|---|
| | **OR (95% Cl)** | ***P*-value** | **OR (95% Cl)** | ***P*-value** |
| Low muscle strength | 0.62 (0.45–0.87) | 0.005 | 0.65 (0.47–0.91) | 0.012 |
| Low muscle mass<br>Height-adjusted | 0.86 (0.70–1.05) | 0.127 | 0.85 (0.70–1.05) | 0.125 |
| BMI-adjusted | 0.79 (0.64–0.98) | 0.029 | 0.78 (0.63–0.97) | 0.024 |
| Sarcopenia<br>Height-adjusted | 0.69 (0.46–1.05) | 0.081 | 0.75 (0.50–1.14) | 0.176 |
| BMI-adjusted | 0.78 (0.53–1.17) | 0.223 | 0.83 (0.56–1.25) | 0.364 |

BMI, body mass index; ASM, appendicular skeletal muscle mass; BMI, body mass index; *ilr*, isometric log-ratio; OR, odds ratio; CI, confidence interval. Height-adjusted and BMI-adjusted indicate that low muscle mass and sarcopenia were defined using height-adjusted and BMI-adjusted ASM, respectively. All models were fitted using Firth's penalized logistic regression. Both primary and sensitivity models were re-estimated among participants with complete data for all covariates included in the sensitivity models. Primary models were adjusted for age and sex, and sensitivity models were additionally adjusted for smoking status, alcohol consumption, educational attainment, marital status, and treatment for hypertension, diabetes, and dyslipidemia.
